# Application of flavonoid compounds suppresses the cotton aphid, *Aphis gossypii*

**DOI:** 10.3389/fpls.2025.1545499

**Published:** 2025-04-03

**Authors:** Ying Zhang, Xinhang Wang, Zhipeng Bian, Chenchen Se, Guoqing Yang, Yanhui Lu

**Affiliations:** ^1^ College of Plant Protection, Yangzhou University, Yangzhou, China; ^2^ State Key Laboratory for Biology of Plant Diseases and Insect Pests, Institute of Plant Protection, Chinese Academy of Agricultural Sciences, Beijing, China; ^3^ Western Agricultural Research Center, Chinese Academy of Agricultural Sciences, Changji, China

**Keywords:** *Aphis gossypii*, flavonoids, antifeedant activity, population Growth, fecundity, ecofriendly pest control

## Abstract

**Introduction:**

The cotton aphid *Aphis gossypii* is a significant polyphagous crop pest and has evolved a high level of resistance to neonicotinoids and other insecticides. Flavonoids, plant phytonutrients, have shown promise as natural insect deterrents and growth inhibitors. However, comprehensive evaluations of the effects of flavonoids on *A. gossypii* are currently lacking.

**Methods:**

In this study, we first evaluated the effects of seven flavonoids (kaempferol, genistein, daidzein, naringenin, rutin, luteolin, and apigenin) on aphid settling behavior using choice assays, followed by electrical penetration graph (EPG) recordings to assess their influence on feeding activity. We then measured honeydew excretion and conducted life table analysis under laboratory conditions to assess effects on growth and reproduction. Under greenhouse conditions, all seven flavonoids were tested for their inhibitory effects on *A. gossypii* population growth over 12 days. Based on the results, three effective flavonoids were selected for further testing at four concentrations (1×, 2×, 3×, and 4× of 1 μg/μL) to assess dose-dependent effects.

**Results:**

We found that all seven flavonoids significantly deterred aphid settling on host plants. Kaempferol, daidzein, naringenin, rutin, luteolin, and apigenin significantly reduced the total duration of phloem feeding and the proportion of time spent on phloem-related activities. And also, each of seven flavonoids reduced honeydew production compared to controls. In the laboratory, all flavonoids reduced adult longevity and fecundity, and kaempferol, genistein, daidzein, naringenin, luteolin and apigenin also reduced the net reproductive rate (R_0_), intrinsic rate of increase (r_m_), and finite rate of increase (λ). Naringenin, apigenin, and kaempferol significantly inhibited *A. gossypii* population growth in a dose-dependent manner over 12 days.

**Discussion:**

These results demonstrate that the seven flavonoids, especially naringenin, apigenin, and kaempferol tested provided effective management of *A. gossypii* populations by deterring host settling, reducing phloem feeding, honeydew production, and decreasing reproductive rates. This study highlights the potential of flavonoids as eco-friendly control agents against *A. gossypii*.

## 1 Introduction

The cotton aphid, *Aphis gossypii* Glover (Hemiptera: Aphididae), is a small yet highly prolific pest that is widespread globally, affecting cotton and melons, and also a variety of other crops (Gul et al., 2023). By feeding on the sap of host plants, *A. gossypii* causes leaf curling, yellowing, and growth inhibition, which can ultimately lead to plant dwarfing, deformity, or even death. Additionally, the aphid secretes honeydew, promoting the growth of sooty mold, and serves as a vector for various plant pathogens, resulting in significant economic losses in agricultural systems (Ji et al., 2021; Nalam et al., 2019; Jayasinghe et al., 2022). Currently, insecticide application is the most common approach used for control of this pest. However, the frequent application and overuse of chemical insecticides have led to the rapid development in *A. gossypii* of resistance to several classes of insecticides, including organophosphorus, pyrethroids, and neonicotinoids (Gore et al., 2013; Koo et al., 2014). Also, these insecticides usually have adverse effects on the environment and non-target organisms, contributing to ecological imbalances in the agro-ecosystem (Aktar et al., 2009). As a result, the overreliance on chemical insecticides has highlighted the urgent need for sustainable alternatives to pest control. In response to these problems, the use of natural insecticides, derived from plants or other natural sources, has increased as an alternative to synthetic chemical insecticides and will provide a more sustainable approach to pest management (Mossa et al., 2018).

Currently, increasing attention is being paid to the use of secondary plant metabolites, which naturally protect many plants from herbivorous insects (Divekar et al., 2022; Campos et al., 2016). Through long-term co-evolution with insects, plants have developed the ability to produce a wide array of secondary metabolites (Bruce, 2015). Over 50,000 secondary metabolites have been identified in the plant kingdom, mainly in the form of terpenes, alkaloids, and flavonoids (Alseekh and Fernie, 2023; Nyamwihura and Ifedayo, 2022). These metabolites, while not essential for normal plant growth, play crucial roles in plant defense using mechanisms such as repellency, antifeedant activity, and inhibition of insect reproduction or other life processes (Ukoroije and Otayor, 2020). They also significantly influence the host plant selection process of herbivorous insects (Wang and Qin, 2007). For example, azadirachtin, a tetranortriterpenoid from the neem seed of the Indian neem tree (*Azadirachta indica* A. Juss (Meliaceae)), is a highly successful botanical insecticide globally. It acts as a powerful antifeedant against 413 insect species and is non-toxic to beneficial biocontrol agents and mammals (Kilani et al., 2021; Pickett et al., 1994). In many plants, such defense compounds are induced by the feeding of pest insects. For example, feeding of *Tetranychus urticae* (Koch) causes its host plant *Phaseolus vulgaris* (L.) to release volatile secondary metabolites that deter further feeding of the two-spotted spider mite (Dicke et al., 1990). In many plants, such defense compounds may be either pre-formed or induced and provide resistance to various pests. For example, the host plants of *Lymantria dispar* (L.) use tannins as defense compounds, and laboratory studies of larvae fed a tannin-enriched artificial diet experience life cycle disruptions, prolonged development, reduced larval body mass, and increased mortality (Wang et al., 2014). Despite the vast diversity of secondary plant metabolites, very few have been investigated and put into practical use. Therefore, more basic research and study of the practical applications of such compounds are needed to explore and harness the potential of these natural compounds against target pests.

Flavonoids are a group of secondary metabolites that are structurally varied and have a wide-range of biological activities, making them a promising class of natural compounds for development for pest control (Pereira et al., 2024). Flavonoids are known to influence the feeding behavior and growth of various insects, including aphids (Simmonds, 2001). For example, heightened levels of quercetin and luteolin disrupt feeding of black bean aphid, *Aphis fabae* Scopoli (Goławska et al., 2024). Similarly, luteolin and genistein have antifeedant properties that disrupt the feeding of pea aphid, *Acyrthosiphon pisum* Harris (Goławska and Łukasik, 2012). Additionally, apigenin have antifeedant effect on *A. pisum* (Goławska et al., 2014a). *In-vitro* bioassays have shown that phloridzin and dihydroquercetin significantly reduce adult survival and fecundity of *A. gossypi*i (Zhang et al., 2024). The current use of flavonoids as antifeedants is extensive, and they merit further development to produce eco-friendly, novel biopesticides. However, the potential of flavonoids for management of *A. gossypii* has not been studied. Based on these previous studies, this study not only investigates the four flavonoids with established antifeedant activity—apigenin, genistein, luteolin, and quercetin—but also expands the scope by selecting additional compounds based on their distinct flavonoid subclasses. Specifically, we included daidzein, an isoflavone similar to genistein; rutin, a flavonol similar to quercetin; and naringenin, a flavanone. This selection strategy considers the representation of different flavonoid subclasses and aims to further explore their potential in the management of *A. gossypii*.

This study aims to comprehensively evaluate the antifeedant and growth inhibitory activities of seven flavonoids (apigenin, luteolin, quercetin, daidzein, genistein, rutin, and naringenin) against *A. gossypii*. Under laboratory conditions, we investigated the efficacy of these compounds using choice settling assays and electrical penetration graph (EPG) tests. Additionally, we quantified honeydew excretion as an indicator of aphid feeding intensity when consuming cotton leaves treated with tested flavonoids. We also investigated the effects of these seven flavonoids on the growth, development, and reproductive capacity of *A. gossypii*. Under greenhouse conditions, we assessed the inhibitory effects of these compounds on the population growth of *A. gossypii*, providing scientific evidence supporting the development of new environmentally friendly control materials for *A. gossypii*.

## 2 Methods

### 2.1 Aphid rearing

*Aphis gossypii* used in our study were collected in 2022 from cotton fields at the Langfang Experiment Station of the Chinese Academy of Agricultural Sciences (CAAS) (39.53°N, 116.70°E) in Hebei Province, China. The sampled cotton field had been maintained without insecticide applications for at least one growing season prior to aphid collection. The aphid population was reared on potted cotton plants at the 4- to 5-leaf stage in a climate-controlled growth chamber set to 26 ± 1 °C, 50 ± 5% relative humidity (RH), and a photoperiod of 16:8 (L:D) hours. All subsequent bioassays were conducted under these same controlled conditions, with observations initiated between 10:00 and 11:00 am to ensure consistency.

### 2.2 Sources of test chemicals

**Table 1** provides details on the purity and sources of the seven compounds and one solvent (ethyl alcohol) used in our study. The preparation and application of these compounds followed the procedures described by Girardi et al. and Powell et al (Girardi et al., 2023; Powell et al., 1997). Specifically, each compound was prepared in 70% ethanol at a concentration of 1 µg/µL. For all compounds, an application volume of 0.01 mL/cm² was evenly distributed on the abaxial (lower) leaf surface using a fine brush. As a control, surfaces of leaves were treated with 70% ethanol, which was the solvent used for the test compounds, using the same volume as for the treatments. Importantly, previous studies have shown that the application of ethanol does not impact aphid probing behavior or aphid assessment of plant condition (Zapata et al., 2010), which was confirmed in our preliminary tests, in which ethanol treatment alone did not affect the aphids or the health of the plants.

**Table 1:** Flavonoids used as treatments: source and purity.

Tested compounds	Structural formula	CAS	Purity (%)	Manufacturer
Kaempferol	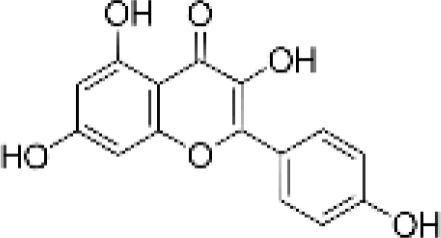	520-18-3	98%	InnoChem Science &Technology Co., Ltd. (Beijing, China)
Genistein	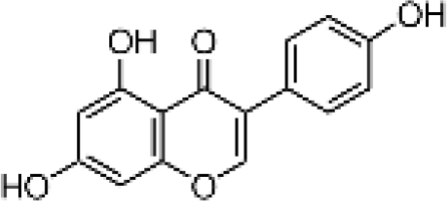	446-72-0	98%	InnoChem Science &Technology Co., Ltd. (Beijing, China)
Daidzein	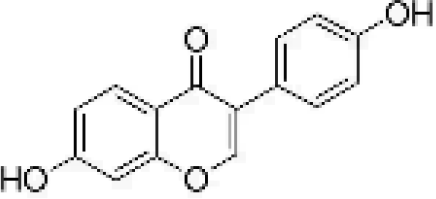	486-66-8	98%	InnoChem Science &Technology Co., Ltd. (Beijing, China)
Naringenin	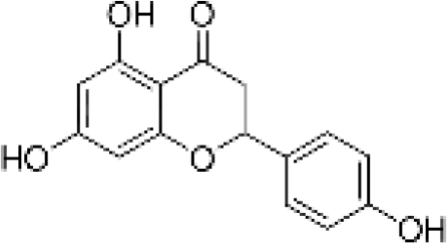	480-41-1	97%	InnoChem Science &Technology Co., Ltd. (Beijing, China)
Rutin	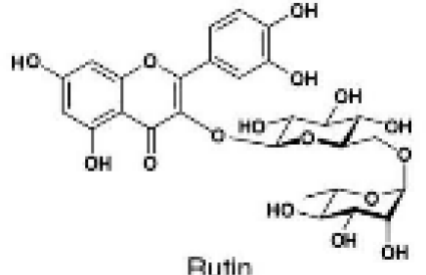	153-18-4	96%	InnoChem Science &Technology Co., Ltd. (Beijing, China)
Luteolin	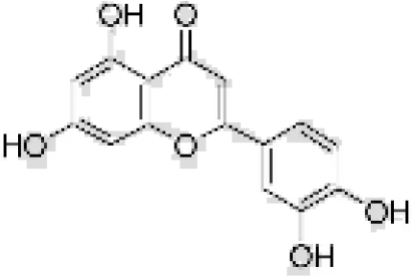	491-70-3	97%	InnoChem Science &Technology Co., Ltd. (Beijing, China)
Apigenin	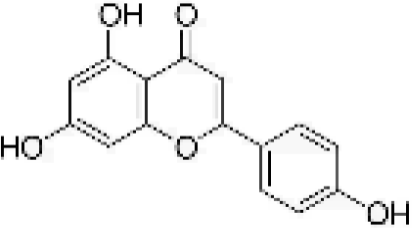	520-36-5	97%	InnoChem Science &Technology Co., Ltd. (Beijing, China)
Ethyl alcohol	—	64-17-5	70%	Sigma-Aldrich, St. Louis, MO.

### 2.3 Exp. #1. Aphid-settling deterrent activity

This experiment used a bioassay to evaluate the host preference of aphids at the leaf level. Aphids settle on a plant only when they recognize it as a suitable food source (Powell et al., 2006). Consequently, the number of aphids that settle on a given substrate serves as a reliable indicator of its suitability. The settling behavior of *A. gossypii* was investigated using a modified “half-leaf test” procedure (Dancewicz et al., 2020). Cotton leaves were cut and placed in a 9 cm diameter petri dish, with the leaves’ lower surfaces facing upward. To prevent desiccation, a water-soaked filter paper (moistened until fully saturated but not dripping) was positioned between the cotton leaves and the petri dish. Compounds were evenly applied to one half of each leaf at a concentration of 1 µg/µL, using a fine brush to ensure uniform coverage. The other side of the midrib was coated with 70% ethanol as a control. There were ten replicates, each consisting of 30 viviparous apterous females. The aphids were placed on the main vein of the leaf, allowing them to choose between equal areas of treated versus control surface. Aphids on treated and untreated leaf surfaces were counted at 1, 2, and 24-hour intervals after the start of the experiment. These observations were made under consistent lighting conditions to avoid influencing aphid behavior.

### 2.4 Exp. #2. Inhibition by flavonoid compounds of aphid-feeding behaviors

Aphid probing behavior was monitored via a technique for electronic recording of aphid probing in plant tissues known as EPG (Tjallingii and Esch, 1993). To record aphid feeding behaviors, a copper electrode connected to a current generator was implanted in the plant tissue, and another electrode was attached to an aphid with a gold wire affixed with conductive silver glue. These electrodes were then connected to an amplifier and computer (EPG Systems, Wageningen, The Netherlands) to detect and record the signal. The probing behavior of apterous females was continuously monitored for 8 hours using an eight-channel DC EPG recorder, until 10 or more completed replicates had been recorded. Incomplete recordings (feeding bouts ending before 8 h) were excluded. Stylet penetration into plant tissue created electrical currents of several different wave types when visualized in an electrical penetration graph. Wave types recognized were those for non-penetration (NP) of plant tissue, stylet penetration initiation (C), short intracellular penetrations (pd), saliva secretion into phloem (E1), phloem sap ingestion (E2), xylem sap ingestion (G), and mechanical penetration difficulties (F). EPG data were annotated using Stylet+ software (Wageningen Agricultural University, Wageningen, The Netherlands).

Each aphid was given access to a freshly prepared plant on which one leaf was treated with one of the test compounds or a solvent (the control). To begin leaf preparation, the cotton leaf was turned over and attached to a piece of cardboard. It was then coated with 1 μg/μL of a test compound using an artist’s brush or for the control leaf was coated with 70% ethanol. The volume of compound used was 500 μL per leaf. Ethanol did not damage the plant tissue. After leaf preparation, aphids were gently placed on the lower surface of individual cotton leaves (choosing the third true leaf) after first being attached to the gold-wire electrode with conductive silver paint. One aphid was placed on each cotton plant for testing. Recordings continued for 8 hours in each session. At the end of each recording session, the cotton leaf and aphid were replaced with new ones. The experiment was conducted in a greenhouse at 26°C, 75% RH, and continuous light. To start recording, the voltage was set to zero. The EPG system amplified the electrical signals, converting them into digital signals that were recorded by Stylet+. The EPG data were annotated using the Stylet+ software, which simultaneously registered the feeding activities of up to eight aphids on eight separate plants.

### 2.5 Exp. #3. Quantification of aphid phloem feeding

The modified honeydew spot area method of Zhang et al. was used to quantify the amount of phloem feeding by recording the amount of honeydew produced in phloem feeding (Zhang et al., 2020). First, filter paper was soaked in a 0.1% solution of bromocresol green and then dried in a 75°C oven to achieve bright orange filter paper. A 2% concentration of agar was then poured into plastic cups (6 cm in diameter), and fresh cotton leaves were placed on the top of the cups. The leaves in the treatment group were coated with 1 μg/μL of the test compound, while the control group leaves were coated with 70% ethanol. Five third-instar aphids, previously starved for 1 hour, were placed in each plastic cup and the aphids allowed to settle on the leaf. The cups were then inverted so that aphids were positioned over the prepared filter paper, allowing honeydew excreted by the aphids to fall onto the paper, which reacted by forming turquoise-blue spots in response to honey dew sugars. After 24 hours, remove the filter paper and measure the area of the blue spots using transparent grid paper (units: mm²). Each small square on the grid represents 1 mm². Count the number of small squares covered by the blue spots. For partial squares, estimate their coverage as one-half or one-third. Finally, sum all the parts to obtain the total area. The experiment was conducted at 25 ± 1°C under natural light conditions, with 18 replicates for each treatment or control group.

### 2.6 Exp. #4. Growth and fecundity of aphids under flavonoid treatments

As another measure of aphid performance of flavonoid-treated plants, we assessed growth and fecundity of aphid cohorts on cotton leaf disks in the laboratory. We followed the approach of Chen and Wang et al (Liu et al., 2021; Wang et al., 2022), but we improved the protocol by using fresh cotton leaves punched into circular pieces with a diameter of 23 mm. Leaf disks were placed upside down in the wells of a 12-well cell culture plate, with each cell containing 1.5 mL of 2% agar. Each leaf disk was coated on the up-facing surface with 1 μg/μL of one test compound, which was applied in 50 μL of solution. One adult aphid was placed in each well, and after 24 hours, one newly produced nymph was retained per well. To prevent escape or movement between aphids, each plate was covered with a lid ventilated with nylon mesh. Every 2 days, the leaf disks were replaced and the test compounds were applied to the new disks. The control group was treated with 70% ethanol. Daily observations were made, recording nymphal molting times, nymphal survival, daily production of nymphs by adults, and daily adult aphid survival, until death. Each treatment group consisted of 50 aphids.

### 2.7 Exp. #5. Population growth of aphids under greenhouse conditions

To further assess the effects of the tested compounds on aphid population growth under more natural conditions, we assessed growth of aphid cohorts established on potted plants held under greenhouse conditions. This experiment consisted of 8 treatments (7 treatment groups and 1 control group), each comprising 10 replicates, with each replicate containing 3 cotton seedlings. Three cotton seedlings were placed in a cage, and approximately 100 aphids were inoculated into each cage. The temperature was maintained at 25 ± 2°C, and a relative humidity at 70 ± 5%. After colonization of plants with aphids (Day 0), the exact number of aphids in each cage was recorded. Following this initial assessment, a small spray bottle was used to apply either the test compounds or 70% ethanol to each cotton seedling. The treatment groups received 1 µg/µL of different flavonoids per plant, while the control group received an equivalent amount of ethanol. Each cotton leaf was evenly sprayed on both sides to ensure uniform application across the entire plant. Aphid numbers on each replicate were recorded on Days 0, 3, 6, 9, 12, and 15.

The three most effective compounds from the initial screening were further tested at 1×, 2×, 3×, and 4× concentrations (based on a 1 µg/µL base concentration) on cotton plants colonized by 100 aphids. The experimental method was the same as described above.

### 2.8 Statistical analysis

Statistical analyses were performed using Microsoft Excel (Microsoft, Redmond, WA, USA) and SPSS v.26.0 software (IBM Corporation, Armonk, NY, USA). Graphs were created using GraphPad Prism 8.0 (GraphPad Software, La Jolla, CA, USA).

For the aphid-settling deterrent activity bioassay (Exp. #1), we compared the number of aphids that settled on treated leaves with those settled on control leaves at 1, 2, 8, and 24 hours after treatment using a Student’s t-test. Before performing the t-test, we checked the normality assumption using the Shapiro-Wilk test. If aphids significantly preferred the leaf treated with the test compound (*P*< 0.05), we described it as having attractant properties. Conversely, if aphids settled mainly on the control leaf (*P*< 0.05), the compound was considered a deterrent. The relative index of deterrence (DI) was calculated as DI = (C – T)/(C + T), where C is the number of aphids settled on the control leaf, and T is the number of aphids settled on the treated leaf. The DI value ranges from −1 (indicating a strong attractant) to 1 (indicating a potent deterrent) (Grudniewska et al., 2015).

For the feeding behavior bioassay (Exp. #2), the honeydew excretion bioassay (Exp. #3), and the greenhouse experiment population growth trial (Ex. #5), we used one-way analyses of variance (ANOVAs) to assess the effects of components on aphids. Prior to performing ANOVA, we assessed the normality assumption using the Shapiro-Wilk test. Tukey’s HSD (honestly significant difference) test was applied for *post-hoc* comparisons to determine statistically significant differences between flavonoid treatments at a significance level of *P*< 0.05.

For Exp. #4 (population growth on leaf discs), in which we recorded aphid development, reproduction rate, and population parameters, we analyzed the data using the age-stage hermaphroditic life table theory with the TWOSEX-MS Chart statistical software (Chi, 1988; Chi and Liu, 1985). Standard errors for nymphal developmental duration, adult aphid longevity, reproduction, and population parameters were calculated using the bootstrap method within the software, with a bootstrap count of 100,000. Differences between treatments were evaluated using the software’s paired bootstrap test (*P*< 0.05).

For the greenhouse experiment (Exp. 5), the growth rate of aphid populations (r) was computed as follows:


$$r=\frac{\left({\text{N}}_{\text{t}}-{\text{N}}_{0}\right)}{{\text{N}}_{0}}$$

where N_t_ is the number of aphids at day t, and N_0_ is the number of aphids at day 0.

The results were visualized in a bubble plot, where the color of each bubble represents the sign of the growth rate (positive or negative), with red indicating a positive growth rate and gray indicating a zero or negative growth rate. The size of each bubble reflects the absolute value of the growth rate, with larger bubbles representing higher growth rates. The statistical analyses were conducted using R software (version 4.0.5), and visualizations were created with the ggplot2 package (Schloerke et al., 2020).

## 3 Results

### 3.1 Exp. #1. Effects of seven flavonoids on aphid settling behavior

The aphid settling bioassay investigated host preferences of aphids under semi-natural conditions. Aphids were observed only to settle on plants that were accepted as a food source, making the number of settled aphids a reliable indicator of a plant’s host suitability. In Exp. #1, significantly fewer aphids (*P*< 0.01) settled on cotton leaves treated with flavonoid compounds compared to the control leaves treated with 70% ethanol (**Table 2**). The deterrent effects were observed as early as one hour after the aphids encountered the treated leaves and persisted for at least 24 hours. The potency and durability of these deterrent effects were high across all tested flavonoids. Specifically, the deterrence indices (DI) exceeded 30% for kaempferol, genistein, luteolin, and apigenin, indicating a strong deterrent effect on *A. gossypii* settling activity.

**Table 2:** Effect of seven flavonoids on settling of *Aphis gossypii*.

Flavonoids tested		Number of aphids			
		1 h	2 h	8 h	24 h
Kaempferol	test	8.90 ± 1.08	9.40 ± 1.27	8.20 ± 0.94	6.30± 0.78
	control	17.30 ± 1.07	17.30 ± 1.16	15.00 ± 0.76	11.30 ± 1.39
	*P*	<0.001***	<0.001***	<0.001***	0.0057**
	DI	0.32	0.30	0.30	0.28
Genistein	test	7.80 ± 0.47	8.30 ± 0.58	8.20 ± 0.83	3.50 ± 0.78
	control	16.00 ± 0.89	15.10 ± 1.24	15.90 ± 0.89	7.30 ± 1.40
	*P*	<0.001***	<0.001***	<0.001***	0.02892*
	DI	0.34	0.29	0.32	0.35
Daidzein	test	8.10 ± 0.69	8.20 ± 0.96	8.60 ± 0.70	7.60 ± 0.67
	control	16.50 ± 0.56	14.50 ± 1.00	15.00 ± 0.80	11.40 ± 1.12
	*P*	<0.001***	<0.001***	<0.001***	0.0092**
	DI	0.34	0.28	0.27	0.20
Naringenin	test	9.50 ± 0.69	8.30 ± 0.73	8.80 ± 0.47	7.00 ± 0.82
	control	15.50 ± 0.76	13.50 ± 0.99	14.50 ± 0.93	11.10 ± 1.23
	*P*	<0.001***	<0.001***	<0.001***	0.0126*
	DI	0.24	0.24	0.23	0.23
Rutin	test	9.70 ± 0.97	9.30 ± 0.79	9.50 ± 0.92	6.70 ± 0.60
	control	17.60 ± 0.90	16.20 ± 0.94	15.10 ± 1.02	13.90 ± 1.10
	*P*	<0.001***	<0.001***	<0.001***	<0.001***
	DI	0.29	0.27	0.23	0.35
Luteolin	test	7.30 ± 0.99	6.90 ± 0.99	5.40 ± 0.85	3.70 ± 0.50
	control	17.50 ± 0.75	17.00 ± 0.71	13.20 ± 0.99	7.50 ± 1.31
	*P*	<0.001***	<0.001***	<0.001***	0.0143*
	DI	0.41	0.42	0.42	0.34
Apigenin	test	8.90 ± 0.64	8.70 ± 0.56	8.60± 0.56	6.30 ± 0.94
	control	17.50 ± 0.64	16.10 ± 0.74	17.90 ± 0.77	12.80 ± 1.67
	*P*	<0.001***	<0.001***	<0.001***	0.0032**
	DI	0.33	0.30	0.35	0.34

The numbers for test and control indicate the mean number of aphids (± SD) that settled on the treated leaves (test) or on the leaves treated with 70% alcohol (control). Student *t*-tests at *P* = 0.05 were used to compare the number of aphids on treated vs. control leaves at each time point. DI is the deterrent index. ‘*’, ‘**’ and ‘***’ indicate significant differences at the *P*< 0.05 level, *P<* 0.01 and *P*<0.001 levels.

### 3.2 Exp. #2. Inhibition of aphid-feeding behaviors by flavonoid compounds

Given that aphids are phloem feeders, any non-phloem activity (**Table 3**) is time not actually spent feeding on plant nutrients. Using wave form analysis over an 8 h aphid feeding bout on control cotton vs. flavonoid-treated leaves, we found that the durations of non-probing (Np) events such as walking and resting by the test aphids were longer in the presence of two of the seven flavonoids tested. This increase in non-productive behavior was statistically significant only for luteolin (F = 3.845, df = 7, 82, *P* = 0.001). Additionally, the treatment with seven flavonoid compounds significantly prolonged the duration of the C-wave (F = 7.626, df = 7, 82, *P*< 0.001). Further multiple comparison analysis revealed that five of these compounds exhibited statistically significant effects compared to the control group, namely kaempferol (*P*< 0.001), genistein (*P*< 0.001), naringenin (*P* = 0.003), rutin (*P* = 0.008), and luteolin (*P* = 0.027). Furthermore, the flavonoid treatments also significantly increased the frequency of C-wave occurrence (F = 4.074, df = 7, 82, *P*< 0.001). Among them, kaempferol (*P*< 0.001), naringenin (*P* = 0.004), and apigenin (*P* = 0.036) showed significant increases compared to the control group. The duration of short potential drops (pd) was also significantly increased by kaempferol (F = 3.127, df = 7, 82, *P* = 0.001). However, there were no significant differences in the duration of xylem feeding (G waves) or time with mechanical difficulties (F waves) among the treatment and control groups.

**Table 3:** Comparison of electrical penetration graph (EPG) parameters of *Aphis gossypii* during the non-phloem phase in the presence of the seven flavonoids.

EPG parameters	Control (n=12)	Kaempferol (n=11)	Genistein (n=11)	Daidzein (n=10)	Naringenin (n=12)	Rutin (n=12)	Luteolin (n=10)	Apigenin (n=12)
NP	10.65 ± 2.88 b	26.25 ± 4.93 ab	18.05 ± 5.51 b	30.05 ± 9.38 ab	34.10 ± 4.43 ab	15.74 ± 4.48 b	51.61 ± 12.66a	26.71 ± 4.60 ab
Pd	10.78 ± 1.25 b	18.46 ± 0.81 a	15.06 ± 1.13 ab	15.41 ± 1.54 ab	16.21 ± 1.22 ab	15.20 ± 1.14 ab	16.19 ± 1.61 ab	14.70 ± 1.17 ab
C	175.41 ± 18.72 c	276.64 ± 12.65 ab	219.43 ± 16.38 bc	314.74 ± 13.06 a	256.31 ± 15.50 ab	250.67 ± 8.64 ab	245.76 ± 19.69 b	229.24 ± 10.40 bc
Total # (C)	176.50 ± 17.07 b	291.82 ± 9.09 a	249.36 ± 17.76 ab	233.40 ± 16.39 ab	268.67 ± 22.06 a	226.25 ± 15.23 ab	240.00 ± 16.91 ab	251.75 ± 17.36 a
Total duration (G) min	30.96 ± 11.65 ab	26.76 ± 7.06 ab	38.22 ± 11.86 ab	14.70 ± 5.87 b	15.62 ± 7.67 b	32.84 ± 9.77 ab	16.04 ± 7.67 b	61.464 ± 9.90 a
Total duration (F) min.	5.60 ± 3.83 a	5.77 ± 4.07 a	15.28 ± 9.64 a	1.21 ± 1.21 a	8.22 ± 5.60 a	6.69 ± 5.11 a	7.32 ± 3.99 a	4.65 ± 3.58 a

Means ± SE followed by the different letters indicate significant differences among seven flavonoids and the control using the ANOVA and Tukey test for *A. gossypii* at *P*<0.05. EPG waveforms are Np, non-probing/penetration; C, pathway waveform; Pd, potential drop; G, xylem feeding; F, period of mechanical difficulty in stylet penetration. The numbers in parentheses are the numbers of aphids tested for each treatment.

Flavonoids also significantly affected the phloem-feeding behaviors of *A. gossypii* (**Table 4**). The aphids spent significantly less time in phloem ingestion (E2) when exposed to the flavonoids (F = 5.577, df = 7, 82, *P*< 0.001). Moreover, all seven flavonoids significantly reduced the ratio of phloem salivation (E1) to phloem ingestion (E2) (F = 4.038, df = 7, 82, *P* = 0.001). There were no significant effects of flavonoids on the number or duration of E1 events, nor on the number of E2 events. Additionally, the times to first salivary secretion (E1) and first established phloem feeding (E2) were not significantly different among treatments.

**Table 4:** Comparison of electrical penetration graph (EPG) parameters of *Aphis gossypii* in the phloem phase in the presence of the seven flavonoids.

EPG Parameters	Control (n=12)	Kaempferol (n=11)	Genistein (n=11)	Daidzein (n=10)	Naringenin (n=12)	Rutin (n=12)	Luteolin (n=10)	Apigenin (n=12)
Time to first E1 (min)	96.66 ± 26.93 a	73.85 ± 23.92 a	52.70 ± 7.95 a	100.93 ± 21.02 a	78.76 ± 21.65 a	98.16 ± 20.74 a	89.69 ± 20.53 a	58.47 ± 7.42 a
Total duration of E1 (min)	32.03 ± 4.99 a	45.84 ± 5.74 a	46.90 ± 8.95 a	42.29 ± 6.80 a	60.82 ± 10.75 a	48.23 ± 6.70 a	34.88 ± 11.79 a	39.69 ± 6.33 a
Total number of E1	16.42 ± 2.71 a	17.82 ± 1.88 a	17.73 ± 2.59 a	12.90 ± 2.32 a	17.33 ± 1.63a	11.75 ± 2.28 a	13.70 ± 2.00 a	18.50 ± 2.83 a
Time to first E2 (min)	102.07 ± 26.25 a	101.97 ± 27.57 a	82.60 ± 20.25 a	151.39 ± 33.57 a	125.08 ± 33.92 a	165.11 ± 41.31 a	115.42 ± 26.59 a	100.19 ± 23.69 a
Total duration of E2 (min)	211.80 ± 22.10 a	80.28 ± 14.09 b	127.05 ± 26.05 ab	61.60 ± 12.35 b	88.73 ± 16.15 b	104.67 ± 20.72 b	108.20 ± 23.28 b	104.16 ± 16.32 b
Total number of E2	7.83 ± 1.22 a	7.82 ± 1.49 a	8.18 ± 1.44 a	5.50 ± 1.21 a	8.17 ± 1.58 a	4.67 ± 1.16 a	6.40 ± 1.50 a	9.25 ± 1.84 a
Percentage of E1+E2	0.51 ± 0.05 a	0.26 ± 0.03 b	0.36 ± 0.06 ab	0.22 ± 0.03 b	0.31 ± 0.04 b	0.33 ± 0.03 b	0.30 ± 0.07 b	0.30 ± 0.04 b

Means ± SE followed by the different letters indicate significant differences among seven flavonoids and the control using the ANOVA and Tukey test for *A. gossypii* at *P*<0.05. EPG waveforms are E1 = salivary secretion; E2 = phloem sap ingestion. The numbers in parentheses are the numbers of aphids tested for each treatment.

### 3.3 Exp. #3. Quantification of aphid phloem feeding

**Figure 1:**
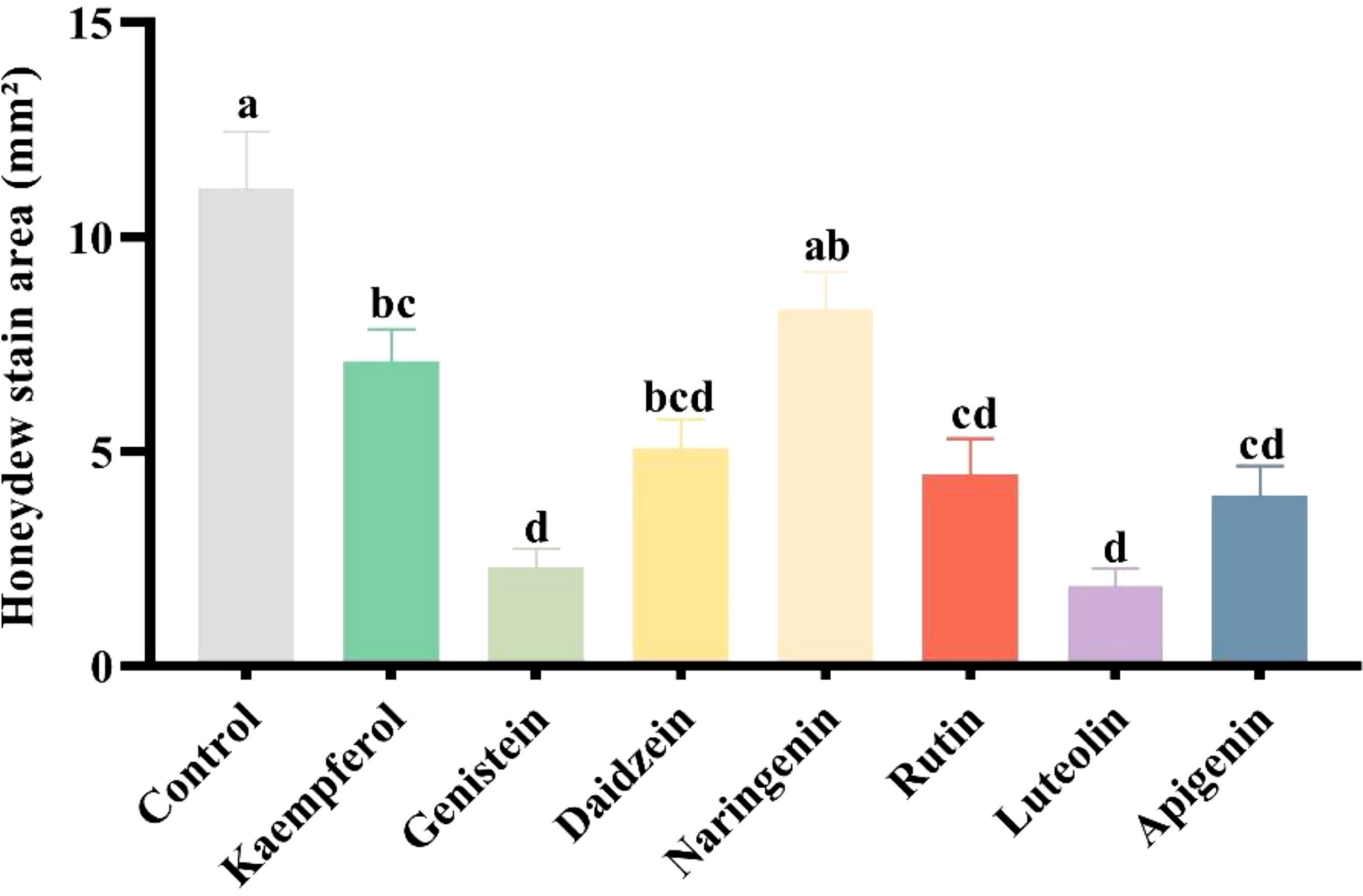
Effect of seven flavonoids on honeydew excretion by *Aphis gossypii*, estimates as the proportion of the filter paper stained by honeydew. Letters above the bars indicate significant differences. The error bars indicate standard error (SE).

In this bioassay, we calculated the quantity of honeydew excreted as an indirect measure of the level of phloem feeding. Because honeydew droplets were dispersed and not overlapping, measurements were not affected by potential aggregations of honeydew. Treatment with all seven flavonoids significantly reduced honeydew excretion by *A. gossypii* compared to the control group (F = 15.787, df = 7, 136, *p*< 0.001) (**Figure 1**).

### 3.4 Exp. #4. Growth and fecundity of aphids under flavonoid treatments

In a leaf-disk, laboratory trial, treatment of foliage with kaempferol significantly prolonged the duration of the third (F = 4.147, df = 7, 352, *P* = 0.002) and fourth instar developmental periods (F = 22.89, df = 7, 352, *P* = 0.006), as well as the overall nymphal period (F = 7.651, df = 7, 352, *P<* 0.001) in *A. gossypii* compared to the control group. However, the other six flavonoid compounds did not significantly affect the developmental period of the *A. gossypii* (**Table 5**), suggesting that kaempferol interferes with *A. gossypii* growth. All seven flavonoid compounds tested had detrimental effects on the longevity and fecundity of *A. gossypii* adults (**Table 6**) in our laboratory, leaf-disk trial. Flavonoids significantly reduced aphid adult longevity (F = 12.295, df = 7, 352, *P*< 0.001) compared to the control. Additionally, the number of reproductive days for adult aphids was significantly reduced (F = 10.751, df = 7, 342, *P*< 0.001). Furthermore, aphid fecundity, measured as the number of offspring produced, was significantly decreased (F = 21.543, df = 7, 352, *P*< 0.001). These results indicate that the flavonoids not only affected the developmental duration of immature stages of *A. gossypii* but also had a substantial impact on the overall lifespan and reproductive capacity.

**Table 5:** Effects of seven flavonoids on growth of immature stages of *Aphis gossypii*.

Flavonoids	Developmental times (d) of each instar				Pre-adult stages
	1^st^	2^nd^	3^rd^	4^th^	
Control	1.50 ± 0.07 a	1.34 ± 0.07 a	1.08 ± 0.04 b	1.24 ± 0.06 b	5.16 ± 0.05 b
Kaempferol	1.46 ± 0.08 a	1.29 ± 0.07 ab	1.39 ± 0.08 a	1.66 ± 0.08 a	5.80 ± 0.14 a
Genistein	1.52 ± 0.08 a	1.11 ± 0.05 ab	1.07 ± 0.04 b	1.34 ± 0.07 ab	5.05 ± 0.10 ab
Daidzein	1.54 ± 0.59 a	1.35 ± 0.07 a	1.28 ± 0.07 ab	1.30 ± 0.07 b	5.48 ± 0.10 ab
Naringenin	1.51 ± 0.07 a	1.11 ± 0.05 b	1.32 ± 0.08 ab	1.45 ± 0.08 ab	5.38 ± 0.11 ab
Rutin	1.42 ± 0.07 a	1.06 ± 0.04 ab	1.13 ± 0.05 b	1.40 ± 0.08 ab	5.00 ± 0.07 ab
Luteolin	1.68 ± 0.08 a	1.21 ± 0.06 ab	1.17 ± 0.06 ab	1.45 ± 0.07 ab	5.51 ± 0.09 ab
Apigenin	1.35 ± 0.09 a	1.22 ± 0.02 ab	1.32 ± 0.08 ab	1.49 ± 0.08 ab	5.38 ± 0.11 ab

Means ± SE followed by different letters indicate significant differences among flavonoids and the control using the ANOVA and Tukey test for *A. gossypii* at *P<*0.05.

**Table 6:** Effects of seven flavonoids on the longevity and fecundity of *Aphis gossypii*.

Flavonoids	Adult longevity (d)	No. oviposition days (d)	Fecundity (individuals per adult)
Control	28.84 ± 0.52 a	13.76 ± 0.21a	68.06 ± 0.82 a
Kaempferol	19.51 ± 1.16 cd	8.79 ± 0.76 d	30.02 ± 3.82 d
Genistein	23.36 ± 0.83 bc	11.67 ± 0.50 bc	45.14 ± 2.95 b
Daidzein	24.30 ± 0.94 b	11.35 ± 0.46 bc	48.72 ± 2.10 b
Naringenin	22.15 ± 0.82 bcd	10.19 ± 0.50 cd	42.49 ± 2.66 bc
Rutin	22.00 ± 0.76 bcd	11.30 ± 0.38 bc	47.98 ± 1.98 b
Luteolin	23.77 ± 0.85 b	12.21 ± 0.44 b	52.81 ± 1.85 b
Apigenin	18.70 ± 1.32 d	8.88 ± 0.83 d	31.35 ± 4.28 cd

Means ± SE followed by different letters indicate significant differences between flavonoids and the control using the ANOVA and Tukey test for *A. gossypii* at *P<*0.05.

The effects of the seven flavonoids tested had significantly varied effects on the population parameters of *A. gossypii* (**Table 7**). Compared to the control, all flavonoids substantially reduced the aphid’s net reproductive rate (R_0_). Kaempferol and apigenin resulted in the lowest R_0_ values, which were significantly lower than the control. The mean generation time (T) varied s among treatments, with kaempferol, daidzein, and luteolin being significantly greater than the control. Except for rutin, the intrinsic rates of increase (rm) and the finite rates of increase (λ) of the aphids feeding of leaves treated with the other six flavonoids - were significantly reduced compared to the control.

**Table 7:** Effects of seven flavonoids on the life table parameters of *Aphis gossypii*.

Flavonoids	R_0_	T(d)	r_m_	λ
Control	68.06 ± 0.81 a	9.47 ± 0.08 bc	0.4457 ± 0.0038 a	1.5616 ± 0.0059 a
Kaempferol	24.62 ± 3.49 d	9.96 ± 0.19 a	0.3216 ± 0.0145 d	1.3793 ± 0.0200 c
Genistein	39.72 ± 3.29 c	9.28 ± 0.14 c	0.3968 ± 0.0111 bc	1.4870 ± 0.0165 b
Daidzein	44.82 ± 2.68 bc	9.90 ± 0.13 a	0.3842 ± 0.0079 c	1.4684 ± 0.0115 b
Naringenin	39.94 ± 2.87 c	9.28 ± 0.14 c	0.3976 ± 0.0101 bc	1.4882 ± 0.0150 b
Rutin	46.06 ± 2.30 bc	8.90 ± 0.12 d	0.4301 ± 0.0081 a	1.5374 ± 0.0124 a
Luteolin	49.64 ± 2.47 b	10.05 ± 0.12 a	0.3884 ± 0.0065 c	1.4746 ± 0.0096 b
Apigenin	23.20 ± 3.69 d	9.75 ± 0.14 ab	0.3226 ± 0.0170 d	1.3808 ± 0.0234 c

Means ± SE followed by different letters indicate significant differences between flavonoids and the control using the ANOVA and Tukey test for *A. gossypii* at *P*<0.05.

### 3.5 Exp. #5. Population growth of aphids under greenhouse conditions in response to flavonoid treatments

**Figure 2:**
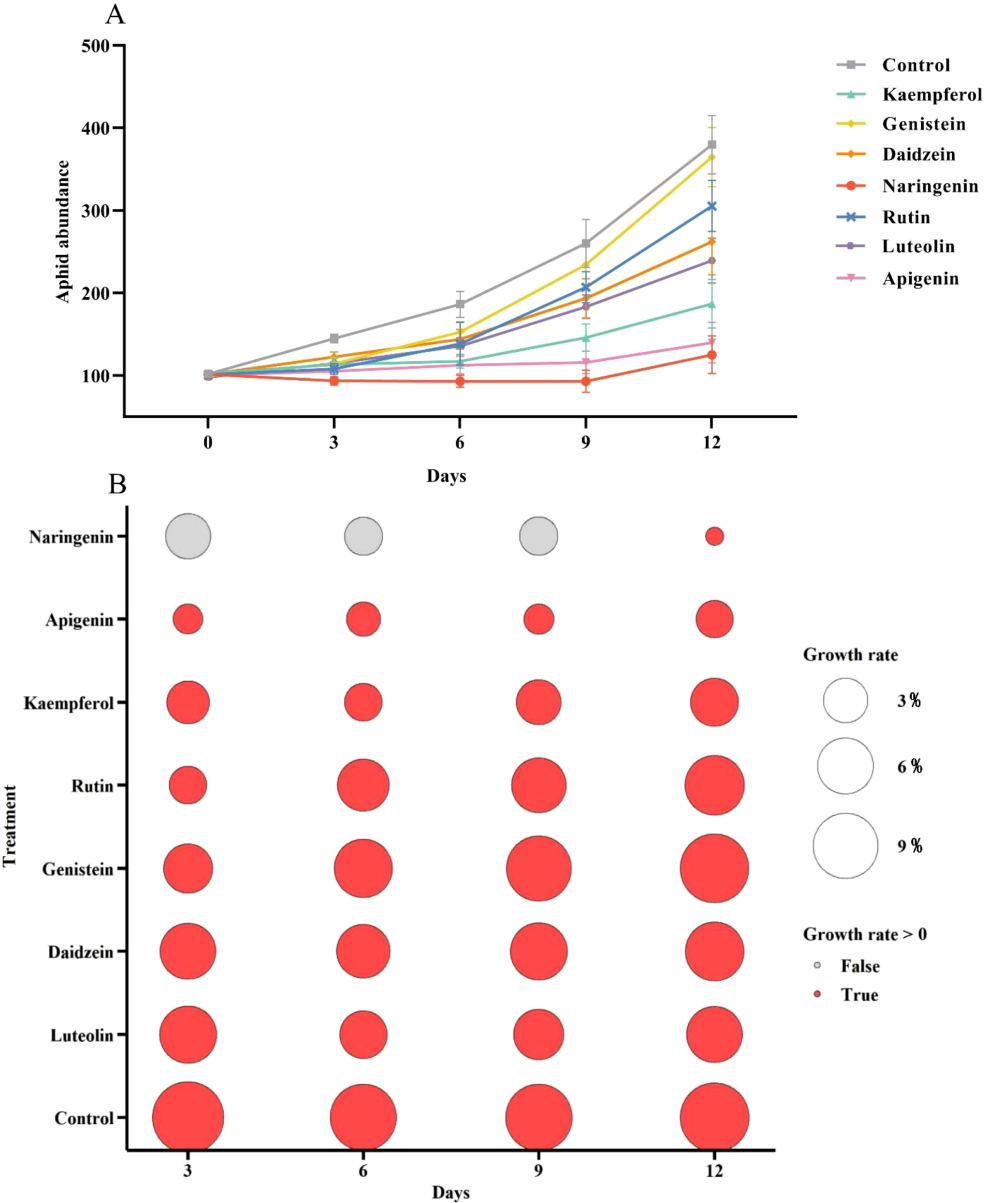
Population abundance and growth rate of *Aphis gossypii* feeding on plants treated with seven flavonoids at a concentration of 1 µg/µL. **(A)** Aphid population abundance and **(B)** population growth rate were recorded following treatment with kaempferol, genistein, daidzein, naringenin, rutin, luteolin, and apigenin. Gray and red circles in panel B indicate growth rates ≤0 and > 0, respectively. The size of the circles represents the growth rate magnitude. Error bars represent standard error (SE).

Beginning with the same initial aphid numbers, treatment with flavonoid compounds at a concentration of 1 µg/µL resulted in a continuous increase in aphid abundance in the control group over 12 days, while the rate of aphid population growth was significantly slower in the flavonoid-treated groups. Among the flavonoids tested, kaempferol, naringenin, and apigenin treatments resulted in the lowest aphid abundance (**Figure 2A**). Subsequently, we analyzed the population growth rates of *A. gossypii* for the 12 days across different treatment groups (**Figure 2B**). Red bubbles indicate positive growth rates, while gray bubbles represent zero or negative rates. The size of each bubble corresponds to the growth rate’s absolute value. Treatments with apigenin, naringenin, and kaempferol significantly reduced *A. gossypii* growth rates, with apigenin showing negative rates (gray bubbles) on days 3, 6, and 9, indicating strong inhibition. In contrast, the control group maintained positive growth rates throughout the experiment, with large bubbles reflecting rapid population expansion. Overall, the seven flavonoid compounds at 1 µg/µL demonstrated inhibitory effects on *A. gossypii* growth, with naringenin, apigenin, and kaempferol being the most effective.

**Figure 3:**
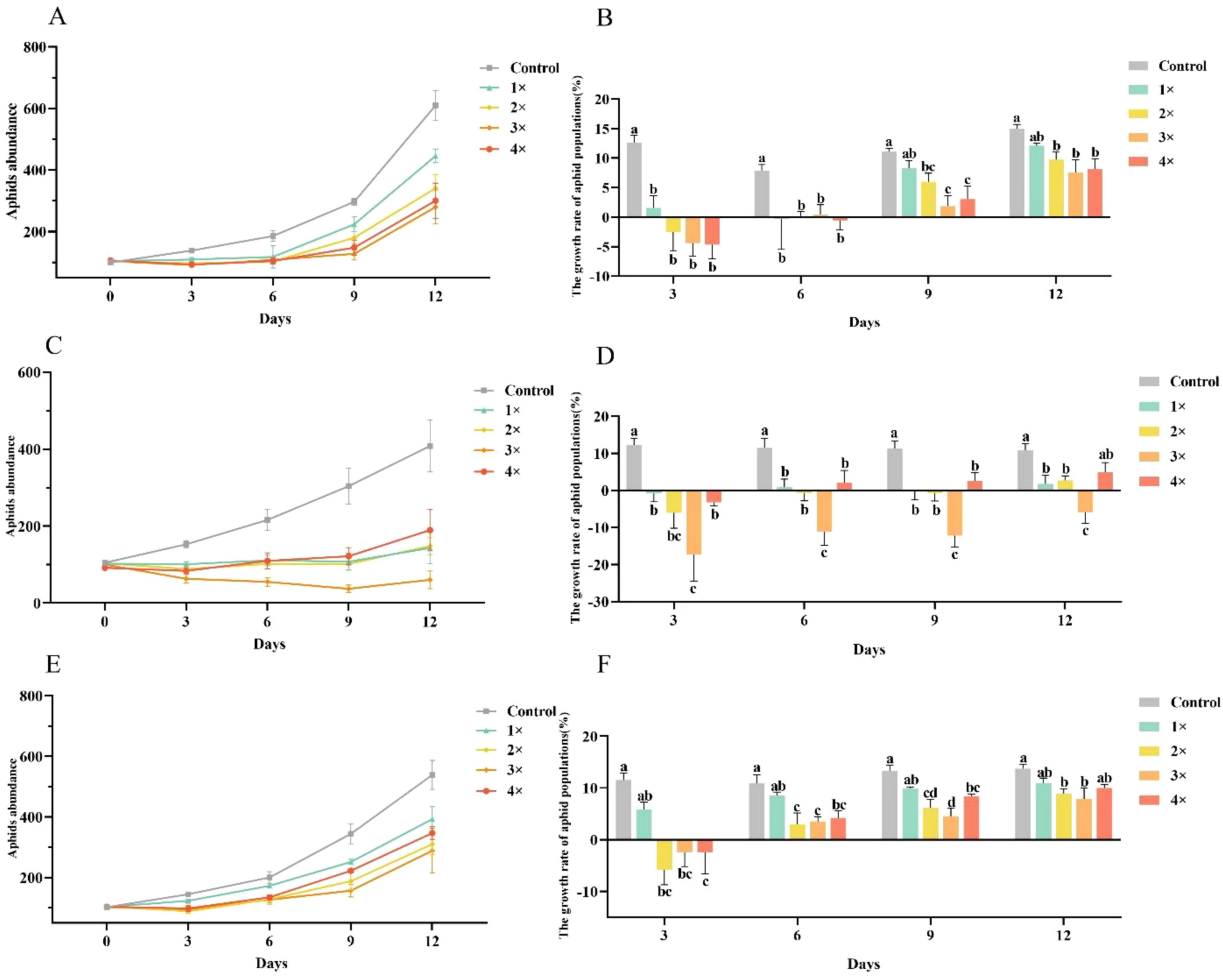
Effects of kaempferol, naringenin, and apigenin on *Aphis gossypii* population dynamics and growth rate. **(A, B)** Effects of kaempferol on aphid abundance and growth rate at 1×, 2×, 3×, and 4× (1 µg/µL base concentration). **(C, D)** Effects of naringenin on aphid abundance and growth rate at the same concentration multiples. **(E, F)** Effects of apigenin on aphid abundance and growth rate at varying multiples of 1 µg/µL. Data points represent mean values ± SE. Letters above the bars indicate statistically significant differences between treatment groups at each time point (p< 0.05).

Further validation with the three most effective flavonoids (naringenin, apigenin and kaempferol) at 1, 2, 3, and 4 µg/µL showed dose-dependent inhibition of *A. gossypii* population dynamics and growth rates (**Figure 3**). From day 3 to day 12, all treatment groups had consistently lower populations than the control (**Figures 3A, C, E**). In the kaempferol group, aphid population growth was significantly lower than that of the control group on days 3 (F = 10.01, df = 4, 15, *P*< 0.001) and 6 (F = 4.53, df = 4, 15, *P* = 0.013). By days 9 and 12, all concentrations significantly reduced the aphid population growth rate (F = 5.922, df = 4, 15, P = 0.005; F = 4.705, df = 4, 15, P = 0.012), with the 2, 3, and 4 µg/µL treatments showing significant differences compared to the control group (**Figure 3B**). In the naringenin group, aphid population growth was significantly lower than that of the control group at all tested concentrations across days 3, 6, 9, and 12 (F = 7.317, df = 4, 15, *P* = 0.002; F = 8.040, df = 4, 15, *P* = 0.001; F = 12.271, df = 4, 15, *P*< 0.001; F = 7.042, df = 4, 15, *P* = 0.002, respectively) (**Figure 3D**). Similarly, in the apigenin group, aphid population growth in the 2, 3, and 4 µg/µL treatments was significantly lower than that of the control group on days 3, 6, 9, and 12 (F = 6.928, df = 4, 15, *P* = 0.002; F = 5.868, df = 4, 15, *P* = 0.005; F = 8.731, df = 4, 15, *P* = 0.001; F = 3.486, df = 4, 15, *P* = 0.033, respectively) (**Figure 3F**). Notably, naringenin exhibited the most significant inhibitory effect, consistent with the previous screening results, further demonstrating its potential for controlling *A. gossypii* populations.

## 4 Discussion

Plants have evolved many secondary metabolites to protect against pests and pathogens. Application of these compounds with feeding deterrent and growth inhibitory properties in insect pest management programs has received much attention in recent years because they are important mediators of plant–insect interactions and can strongly affect insect behavior (Zaynab et al., 2018; El-Aswad et al., 2004). In this study, we selected seven flavonoid secondary metabolites and confirmed that they exhibit both antifeedant effects and growth-reducing properties against *A. gossypii*. These seven flavonoids reduced settling of host plants and inhibited aphid feeding, as shown by reduced deposition of honeydew. These compounds also reduced the reproductive capacity and intrinsic growth rate of *A. gossypii*.

Flavonoids have demonstrated strong antifeedant effects against several other aphids. These previous studies primarily focused on aphid probing behavior. For instance, luteolin prolongs the time to the aphid’s first active ingestion bout, the duration of the first active ingestion, and the average time spent on this activity in *Aphis fabae* Scopoli (Goławska et al., 2024). Additionally, daidzein and kaempferol hindered aphids’ ability to locate sap-transporting vessels (Stec et al., 2021a). Apigenin, on the other hand, increased the duration of probes in non-phloem tissues of *A. pisum* (Stec et al., 2021b). Research on the inhibitory effects of aphid feeding behavior highlights the significance of compelling aphids to spend more time searching for suitable feeding sites. This extended search time can reduce the overall damage they cause to plants. In our present study, we also found that luteolin had a similar effect. Furthermore, our EPG results showed that in the presence of the six flavonoids (kaempferol, daidzein, naringenin, rutin, luteolin and apigenin), the time spent feeding (phloem ingestion) decreased. *A. gossypii* primarily rely on plant phloem sap for essential nutrients to support growth and development. The cessation of sap ingestion shortly after feeding initiation may indicate the presence of feeding deterrents (Pompon and Pelletier, 2012). Inhibition of feeding behaviors of herbivorous insects is a complex and multifaceted process (Xue et al., 2022). Types of antifeedant activities have been categorized as “pre-ingestive” “ingestive”, and “post-ingestive” inhibition, depending on where in the feeding process inhibition acts. Mechanisms of deterrence may act separately or in combination, and the impact of compounds depends on the insect species (Frazier and Chyb, 1995), which was also the case in the present study, in which luteolin showed both pre-ingestive and ingestive deterrent activity.

The secretion of honeydew in aphids is an intrinsic consequence of phloem feeding in aphids. During phloem feeding, aphids must continuously produce honeydew. However, honeydew production is minimal before the stylet reaches the phloem or during xylem feeding (Seo et al., 2009). In this study, we observed a significant decrease in honeydew production in aphids treated with the seven flavonoids tested because *A. gossypii* spent less time phloem feeding. This reduction honeydew on plants treated with flavonoids confirms the deterrent effect of flavonoids on *A. gossypii*. Additionally, any reduction in honeydew production also reduces the negative effect of honeydew on crops, including loss of photosynthesis due to physical coverage of foliage by sooty molds (Joschinski and Krauss, 2017).

In our study, flavonoids also significantly reduced *A. gossypii* reproduction (and therefore the intrinsic population growth rate). This aligns with findings from previous research. For instance, Quercetin and rutin significantly disrupt the feeding behavior of aphids, which is a crucial factor in reducing their reproductive success (Ateyyat et al., 2012). Disruptions in feeding behavior likely limit the aphids’ nutrient intake, thereby negatively impacting their reproductive capabilities and overall fitness. This is consistent with our observations, where treated aphids exhibited reduced reproduction rates. Given the reduction we observed in phloem feeding on flavonoid-treated foliage and considering the role of phloem as the key source of nutrients for aphids, we conclude that the negative effects of plant flavonoids on *A. gossypii* development are likely due to a shortage of nutrients resulting from the reduced duration of aphid phloem feeding.

The flavonoid compounds we tested also inhibited the population growth of *A. gossypii* to some extent. Among all the tested flavonoids, naringenin, apigenin, and kaempferol showed the greatest inhibitory effect on population growth. Previous studies support the inhibitory effects of these three compounds on aphids. For instance, increasing the concentrations of the flavonoids naringenin and quercetin in a liquid artificial diet used to feed *A. pisum* significantly increases the aphid’s pre-reproductive period and decreases its fecundity (Goławska et al., 2014b). Apigenin has been found to significantly extend probing duration in non-phloem tissues in *A. pisum*, indirectly suppressing aphid feeding on host plants (Goławska and Łukasik, 2012). When pea plants are infested by pea aphids (*A. pisum*), the levels of kaempferol and other flavonoids in the plants increase significantly, suggesting that these phenolic compounds may play an important role in the plant’s defense mechanism and exert an inhibitory effect on the aphids (Goławska et al., 2008). However, over time, the inhibitory effect gradually weakened. This reduction in efficacy might have been due to the degradation of the flavonoid compounds. Alternatively, *A. gossypii* might have gradually developed some tolerance to these compounds. Furthermore, the natural decline in leaf quality due to age could also have affected our experimental results by making it more difficult detect any additional inhibitory effects.

Our study demonstrated that flavonoids exert significant inhibitory effects on *A. gossypii* by affecting feeding and reproduction. While this study provides insights into the behavioral and physiological impacts of flavonoids, the precise molecular mechanisms remain to be further explored. Previous studies have suggested that antifeedant compounds may exert their effects by interacting with insect gustatory receptors or digestive enzyme. For example, research on *Drosophila* has shown that the gustatory receptor GR33a plays a key role in the perception of antifeedants, as mutants lacking this receptor fail to avoid ingesting various bitter substances (Moon et al., 2009). Antifeedant compounds, such as those found in *Boerhavia diffusa* L. leaf extract, can inhibit essential digestive enzymes in insects, including protease and amylase (Bindhu et al., 2016). This finding suggests that flavonoids, as plant-derived antifeedants, may similarly influence insect feeding behavior by interacting with gustatory receptors and digestive enzymes. Future research utilizing molecular techniques such as gene expression analysis and enzyme activity assays could further elucidate their mechanisms of action, thereby enhancing the application potential of flavonoid-based pest control strategies.

In integrated pest management (IPM) programs, plant-derived flavonoids have emerged as promising natural antifeedants due to their low toxicity, environmental sustainability, and minimal impact on natural enemies. Unlike conventional chemical insecticides such as neonicotinoids (e.g., imidacloprid and thiamethoxam), which act rapidly through direct toxicity, flavonoids primarily inhibit aphid feeding and reproduction, indirectly suppressing population growth over time. While their application alone may not provide immediate control under field conditions, combining them with low-dose (LC50) chemical insecticides could enhance efficacy while reducing synthetic pesticide use. This approach not only improves pest suppression but also helps mitigate adverse effects on non-target organisms and delays insecticide resistance, promoting a more eco-friendly and sustainable pest control solution within an IPM framework. Our study highlights the potential of developing plant-derived flavonoids as novel aphid antifeedants, paving the way for natural, selective pest control alternatives.

Although flavonoid compounds hold great potential for aphid control, their large-scale agricultural application requires overcoming key challenges. Field adaptability remains a concern, as environmental factors such as temperature, humidity, UV exposure, and rainfall may accelerate degradation and reduce efficacy. Additionally, stability limitations affect field persistence, as flavonoids are susceptible to degradation under light and oxygen exposure. Addressing these issues will require innovative formulation strategies, such as microencapsulation and nanocarrier technologies, to enhance photostability and controlled release. As a plant-derived, biodegradable alternative to synthetic pesticides, flavonoids align with the principles of green pest management, making their development crucial for sustainable agriculture. Optimizing application strategies (e.g., determining the minimum effective concentration threshold) will help balance efficacy and economic feasibility for field use. By integrating flavonoids into IPM systems—whether as single botanical antifeedants or in combination with biological control agents and reduced-risk insecticides—these compounds hold great promise for scalable, eco-friendly aphid management. Further research should refine their applicability across diverse cropping systems and enhance formulation technologies to ensure stability, persistence, and cost-effectiveness, ultimately establishing plant-derived flavonoids as a truly green alternative to conventional insecticides.

## 5 Conclusion

In conclusion, we demonstrated that seven flavonoids (kaempferol, genistein, daidzein, naringenin, rutin, luteolin, and apigenin) exhibit significant antifeedant effects against *A. gossypii*. These flavonoids deter aphid settling, reduce the duration of phloem feeding, and reduce the level of honeydew production. Furthermore, they also negatively affect aphid growth and adult fecundity. Under greenhouse conditions, these compounds inhibited aphid population growth, with naringenin, apigenin, and kaempferol showing the highest impacts. These findings suggest that flavonoids are promising agents for aphid control. Future research should focus on conducting field trials and integrating these flavonoid-based antifeedants into comprehensive pest management strategies.

## Data Availability

The raw data supporting the conclusions of this article are available from the corresponding authors upon reasonable request.
